# Apocynin Prevents Vascular Effects Caused by Chronic Exposure to Low Concentrations of Mercury

**DOI:** 10.1371/journal.pone.0055806

**Published:** 2013-02-04

**Authors:** Danize A. Rizzetti, João Guilherme D. Torres, Alyne G. Escobar, Franck M. Peçanha, Francielli W. Santos, Robson L. Puntel, María J. Alonso, Ana M. Briones, Mercedes Salaices, Dalton V. Vassallo, Giulia A. Wiggers

**Affiliations:** 1 Postgraduate Program in Biochemistry, Universidade Federal do Pampa, Uruguaiana, RS, Brazil; 2 Department of Physiological Sciences, Universidade Federal do Espírito Santo, Vitória, ES, Brazil; 3 Department of Biochemistry, Physiology and Molecular Genetics, Universidad Rey Juan Carlos, Alcorcón, Spain; 4 Department of Pharmacology, School of Medicine, Universidad Autónoma de Madrid, Madrid, Spain; Osaka University Graduate School of Medicine, Japan

## Abstract

Mercury increases the risk of cardiovascular disease and oxidative stress and alters vascular reactivity. This metal elicits endothelial dysfunction causing decreased NO bioavailability via increased oxidative stress and contractile prostanoid production. NADPH oxidase is the major source of reactive oxygen species (ROS) in the vasculature. Our aim was to investigate whether treatment with apocynin, an NADPH oxidase inhibitor, prevents the vascular effects caused by chronic intoxication with low concentrations of mercury. Three-month-old male Wistar rats were treated for 30 days with a) intramuscular injections (*i.m.*) of saline; b) HgCl_2_ (*i.m.* 1^st^ dose: 4.6 µg/kg, subsequent doses: 0.07 µg/kg/day); c) Apocynin (1.5 mM in drinking water plus saline *i.m.*); and d) Apocynin plus HgCl_2_. The mercury treatment resulted in 1) an increased aortic vasoconstrictor response to phenylephrine and reduced endothelium-dependent responses to acetylcholine; 2) the increased involvement of ROS and vasoconstrictor prostanoids in response to phenylephrine, whereas the endothelial NO modulation of such responses was reduced; and 3) the reduced activity of aortic superoxide dismutase (SOD) and glutathione peroxidase (GPx) and increased plasma malondialdehyde (MDA) levels. Treatment with apocynin partially prevented the increased phenylephrine responses and reduced the endothelial dysfunction elicited by mercury treatment. In addition, apocynin treatment increased the NO modulation of vasoconstrictor responses and aortic SOD activity and reduced plasma MDA levels without affecting the increased participation of vasoconstrictor prostanoids observed in aortic segments from mercury-treated rats. Conclusions: Mercury increases the vasoconstrictor response to phenylephrine by reducing NO bioavailability and increasing the involvement of ROS and constrictor prostanoids. Apocynin protects the vessel from the deleterious effects caused by NADPH oxidase, but not from those caused by prostanoids, thus demonstrating a two-way action.

## Introduction

Mercury is a heavy metal that affects the activity of several enzymes, ion channels and receptors [1] by binding to the SH groups [2] that are necessary for normal enzyme function. This metal is a public health problem because many populations worldwide are exposed to mercury at levels that exceed the recommended safety guidelines [3]. Despite attempts to control industrial pollution, mercury poisoning still occurs from exposure to methylmercury (MeHg) from fish, ethylmercury from vaccine products, metallic mercury from dental amalgam fillings, cinnabar from Chinese herbal balls, and other sources of domestic mercury contamination [4], [5].

The harmful effects of mercury during its accumulation in humans are mostly due to the excessive release of reactive oxygen species (ROS), the increased lipid peroxidation in the cells and the reduction of antioxidant defenses, thus inactivating important enzymes that are responsible for body's defenses, such as glutathione reductase (GR), glutathione peroxidase (GPx), superoxide dismutase (SOD), catalase (CAT) and GSH in different organs [6], [7]. These effects are capable of damaging the integrity and altering the function of membranes, which can lead to the development of many pathological processes [8]. Because mercury exposure is associated with oxidative stress and endothelial dysfunction, more attention has been paid to its toxic effects on the cardiovascular system and its association with hypertension, carotid atherosclerosis, myocardial infarction, and coronary heart disease [9], [10].

Previous studies from our group have demonstrated that oxidative stress caused by mercury exposure decreases the bioavailability of nitric oxide (NO) and alters the expression of NO synthase (NOS), leading to increased vasoconstriction, the reduction of endothelial vasodilator response and the stimulation of cyclooxygenase (COX)-derived vasoconstrictor prostanoids release [11]–[14]. NADPH oxidase, a multisubunit enzymatic complex, has been considered the major source of ROS in vascular cells [15] and has been suggested to be responsible for the endothelial dysfunction observed in different cardiovascular pathologies [10], [16]. Interestingly, previous studies have also reported that the activation of NADPH oxidase may be associated with exposure to mercury in mesenteric and coronary arteries [12], [14].

Therefore, the aim of this study was to assess whether treatment with the NADPH oxidase inhibitor apocynin can prevent or mitigate the changes caused by chronic exposure to low doses of mercury on a) the endothelial modulation of vasoconstrictor and vasodilator responses in conductance arteries; b) ROS and COX participation in these vascular responses; and c) ROS production and antioxidant activity.

## Materials and Methods

### Animals

Three-month-old male Wistar rats (290–310 g) were obtained from the Central Animal Laboratory of the Federal University of Santa Maria, Rio Grande do Sul, Brazil. During treatment, rats were housed at a constant room temperature, humidity, and light cycle (12:12 h light-dark), free access to tap water and fed with standard chow *ad libitum*. All experiments were conducted in compliance with the guidelines for biomedical research stated by the Brazilian Societies of Experimental Biology and approved by the Ethics Committee on Animal Experimentation of the Escola de Ensino Superior da Santa Casa de Misericórdia de Vitória, EMESCAM, Vitória, Espírito Santo, Brazil (Process Number: 010/2011).

Rats were divided into four groups and treated for 30 days as follows: a) Untreated (saline solution, *i.m.*); b) Mercury (HgCl_2_) - mercury chloride (1^st^ dose 4.6 µg/kg, subsequent doses 0.07 µg/kg/day, *i.m.*, to cover daily loss, using the model described by Wiggers *et al*. [17]); c) Apocynin (Apo) - apocynin (1.5 mM in drinking water plus saline solution, *i.m.*); and d) Apocynin-mercury (ApoHg) - mercury chloride plus apocynin.

### Systolic (SBP) and diastolic (DBP) blood pressure

Rats were anesthetized with urethane (1.2 g/kg, *i.p*.), and after loss of the righting reflex, the carotid artery was cannulated with a polyethylene catheter (PE 10, *Clay-Adams*, NY, USA), filled with saline plus heparin (50 U/ml) to measure arterial pressure. SBP and DBP were measured using a pressure transducer (TSD104A) connected to an amplifier and an acquisition system (MP 150 Biopac Systems, Inc., CA, USA) and were continuously monitored. After a 30-min stabilization period, the SBP and DBP were obtained and recorded.

### Blood collection and reactivity experiments

Rats were submitted to a surgical procedure to expose and puncture the renal artery; blood was subsequently collected to obtain plasma for the biochemical experiments. Thereafter, rats were euthanized by decapitation, and the thoracic aorta was carefully dissected out and cleaned of connective tissue. For reactivity experiments, the thoracic aorta was divided into segments that were 2 mm in length. For isometric tension recording, each aortic segment was set up in an organ bath containing 5 ml of Krebs-Henseleit solution (KHS, in mM: 115 NaCl, 25 NaHCO_3_, 4.7 KCl, 1.2 MgSO_4_ 7H_2_O, 2.5 CaCl_2_, 1.2 KH_2_PO_4_, 11.1 glucose and 0.01 Na_2_EDTA) at 37°C and continuously gassed with a 95%O_2_-5%CO_2_ mixture (pH = 7.4). Two horizontally arranged stainless steel pins (75 µm in diameter) were passed through the lumen: one was fixed to the organ bath wall, and the other was vertically connected to a force-displacement transducer (TSD125BX8 - Biopac Systems, Inc) and a recorder (MP150WSW-SYS - Biopac Systems, Inc).

Aortic segments were subjected to a tension of 1.5 g that was readjusted every 15 min during a 60-min equilibration period before drug administration. Vessels were initially exposed to 75 mM KCl to check their functional integrity, and the presence of endothelium was confirmed by the ability of acetylcholine (10 µM) to relax segments contracted with phenylephrine at a concentration that produces close to 50% of the contraction induced by 75 mM KCl. After 60 min of washout, a single concentration-response curve to phenylephrine (0.01 nM – 300 µM) was performed.

To evaluate the role of the endothelium in the vasoconstrictor response to phenylephrine, some rings had their endothelium removed mechanically, and its absence was confirmed by the inability of acetylcholine to induce relaxation greater than 10% of the previous contraction to phenylephrine. The effects of N_ω_-Nitro-L-arginine methyl ester hydrochloride (L-NAME – 100 µM), indomethacin (1 µM) and apocynin (0.3 mM) were investigated by their addition 30 min before phenylephrine in vessels with intact endothelium. Thus, to evaluate the participation of NO, prostanoids or ROS on phenylephrine responses, the effect of the in vitro addition of those drugs was compared with the control situation (intact endothelium) in the absence or drugs.

To evaluate the relaxation dependent and independent of the endothelium, concentration-response curves were performed with acetylcholine (0.01 nM – 300 µM) and sodium nitroprusside (SNP, 0.01 nM – 300 µM), respectively.

### Lipid peroxidation in plasma

Plasma thiobarbituric acid-reacting substances (TBARS) are lipid peroxidation products that are considered an expression of systemic oxidative stress and are measured as malondialdehyde (MDA) using a colorimetric method, as previously described by Ohkawa *et al*. [18], with modifications. Blood was collected by renal puncture and transferred into tubes containing the anticoagulant EDTA (Wiener Lab, Rosario, Argentina). Plasma was obtained by centrifugation (1500xg, 4°C, 15 min). An aliquot of plasma was incubated with thiobarbituric acid 0.8% (TBA), phosphoric acid buffer 1% (H_3_PO_4_), and sodium dodecil sulphate 0.8% (SDS) at 100°C for 60 min. The color reaction was measured at 532 nm against blanks (Spectrophotometer *Femto 600 S, FEMTO*, São Paulo, Brazil). The results were expressed as nanomoles of MDA per ml of plasma.

### Thiol groups in plasma

The total concentration of thiol groups (SH) was measured using spectrophotometry at 412 nm, according to Ellman's method [19]. An aliquot of blood plasma was mixed with 10% SDS and 1 M phosphate buffer (pH 8), and the absorbance was measured at 412 nm (A0) against blank. Then, 5,5’-dithio-bis-2-nitrobenzoic acid 10 mM (DTNB) was added followed by incubation at 37°C for 60 min. After incubation, the absorbance of the sample was again measured at 412 nm (A1). The result is the difference between A1-A0 and was expressed as nanomoles of thiol groups per ml of plasma.

### Superoxide dismutase (SOD) and glutathione peroxidase (GPx) activity in aortas

SOD activity in aorta homogenate was assayed using spectrophotometry, as described by Misra and Fridovich [20]. This method is based on the capacity of SOD to inhibit the autoxidation of adrenaline to adrenochrome. The color reaction was measured at 480 nm. One unit of enzyme was defined as the amount of enzyme required to inhibit the rate of epinephrine autoxidation by 50% at 26°C. Aortas were washed with ice-cold saline and rapidly homogenized in 50 mM Tris–HCl, pH 7.5 (1∶10, w/v) and centrifuged at 2400x*g* for 15 min at 4°C. The supernatants (S1) were then separated. The S1 was diluted 1∶10 (v/v) for determination of SOD activity on test day and added to a 50 mM glycine buffer (pH 10.3). The enzymatic reaction was started by adding 60 mM epinephrine, and the enzymatic activity was expressed as Units (U) per mg of protein.

GPx activity in aorta tissue was assayed using spectrophotometry using the method of Wendel [21] through the reduced glutathione (GSH)/β tetrasodium salt (NADPH)/glutathione reductase system by the dismutation of H_2_O_2_ at 340 nm. In this assay, the enzyme activity was indirectly measured using an NADPH decay. H_2_O_2_ was decomposed, generating oxidized glutathione (GSSG) from GSH. GSSG was regenerated back to GSH by glutathione reductase present in the assay medium at the expense of NADPH. S1 was added to the GSH/NADPH/glutathione reductase system, and the enzymatic reaction was initiated by adding H_2_O_2_ 4 mM. The enzymatic activity was expressed as nmol NADPH/per min per mg protein. Proteins for both experiments were measured according to Bradford using bovine serum albumin as a standard.

### Drugs and reagents

HgCl_2_, apocynin, phenylephrine hydrochloride, acetylcholine chloride, SNP, urethane, L-NAME and indomethacin were purchased from Sigma-Aldrich (St Louis, MO, USA); heparin was purchased from Roche (São Paulo, SP, Brazil). Salts and reagents, when not specified, were of analytical grade obtained from Sigma and Merk (Darmstadt, Germany).

### Data analysis and statistics

All values are expressed as the mean±SEM of the number of animals used in each experiment. In the vascular reactivity experiments, vasoconstrictor responses were expressed as the% of contraction induced by 75 mM KCl and vasodilator responses as the% of the previous contraction to phenylephrine. To compare the effect of L-NAME, apocynin and indomethacin on the response to phenylephrine in segments from the different groups, some results were expressed as ‘differences of area under the concentration-response curves’ (dAUC) in control and experimental situations. AUCs were calculated from the individual concentration-response curve plots; the differences were expressed as the percentage of the AUC of the corresponding control situation. The results were analyzed using either Student's *t*-test or two-way ANOVA for comparison between groups. When ANOVA showed a significant treatment effect, Bonferronís post hoc test was used to compare individual means. Differences were considered statistically significant at P<0.05.

## Results

No differences in body weight were observed between all groups before and after treatment with mercury, as previously described [17]. (Untreated: 302.4 ± 30.1 g before and 332.7 ± 26.3 g after; HgCl_2_-treated: 309.6 ± 56.8 g before and 346.3 ± 54.2 g after - *t*-test - P>0.05). Apocynin alone or co-treatment with mercury plus apocynin did not change this parameter (ApoHg-treated: 309.0 ± 28.4 g before and 347.4 ± 26.1 g after; Apo-treated: 299.3 ± 46.4 g before and 335.4 ± 36.1 g after - *t*-test - P>0.05).

### Effect of apocynin on systolic and diastolic blood pressure

**Figure 1:**
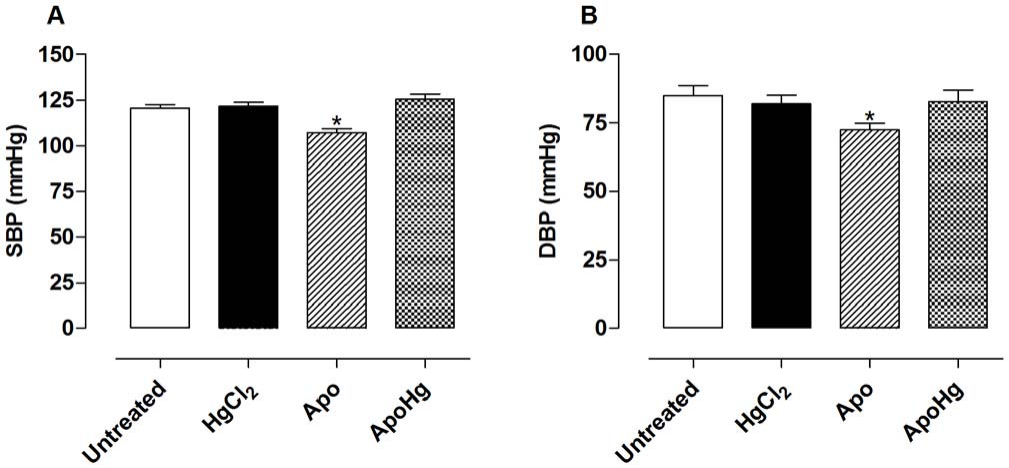
Effect of apocynin treatment on systolic and diastolic blood pressure. Values of (A) systolic (SBP) and (B) diastolic blood pressure (DBP, mmHg) in the aorta of rats untreated (n = 9) or treated with mercury (HgCl_2_, n = 8), apocynin (Apo, n = 9) or apocynin plus mercury (ApoHg, n = 9). The results are expressed as the mean±SEM, *t*-test *P<0.05 *vs*. Untreated.

Chronic treatment with low doses of mercury did not change either SBP or DBP. Apocynin decreased SBP and DBP in untreated rats; however, it was unable to exert this effect in the group treated concomitantly with mercury (Figure 1).

### Apocynin treatment improves endothelium-dependent vasodilator responses and reduces vasoconstrictor responses in mercury-treated rats

**Figure 2:**
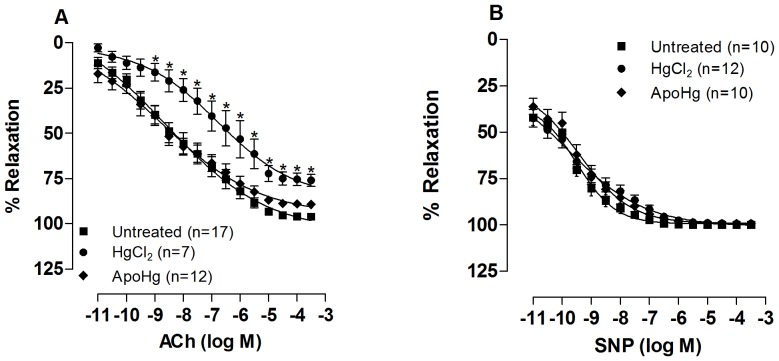
Effect of apocynin treatment on the vascular relaxation response to acetylcholine and sodium nitroprusside. Concentration-response curves to (A) acetylcholine (ACh) and (B) sodium nitroprusside (SNP) in the aortas of rats untreated, treated with mercury (HgCl_2_) or apocynin plus mercury (ApoHg) pre-contracted with phenylephrine. The results (mean±SEM) are expressed as a percentage of the response to phenylephrine. The number of animals used is indicated in parentheses. Two-Way ANOVA *P<0.05.

Exposure to acetylcholine and SNP produced concentration-dependent relaxation in the aortic rings from all groups. As previously described [12], chronic exposure to mercury reduced the vascular response to acetylcholine and co-treatment with apocynin prevented this reduction (Figure 2A, Table 1). SNP responses were similar in untreated, mercury-treated and mercury-treated plus apocynin (Figure 2B, Table 1). Apocynin treatment did not affect the acetylcholine or SNP responses in the absence of mercury (Table 1). These results suggest that chronic exposure to low doses of mercury promoted endothelial dysfunction in the rat aorta, which was prevented by apocynin.

**Table 1:** Effects of apocynin treatment of rats in the absence and in the presence of HgCl_2_ on maximum response (R_max_) and sensitivity (pD2) to acetylcholine and sodium nitroprusside.

	ACh		SNP	
	R_max_	pD2	R_max_	pD2
Untreated	102.2±13.7	-8.1±1.4	100.2±1.6	−9.5±0.5
HgCl_2_	78.3±9.3*	−7.1±1.3	99.7±3.4	−9.0±0.6
Apo	97.1±9.1	−7.9±1.8	95.9±7.3	−8.3±0.3
ApoHg	96.4±8.3^#^	−8.4±1.2	100.4±2.6	−9.1±0.4

Parameters of maximal response (Rmax) and sensitivity (pD2) of the concentration-response curves to acetylcholine (ACh) and sodium nitroprusside (SNP) in aortas from rats untreated, treated with mercury (HgCl_2_), apocynin and apocynin plus mercury (ApoHg) in intact segments (Control). Results are expressed as mean±SEM. R_max_, maximal effect (expressed as a percentage of the previous contraction to phenylephrine) and pD2 expressed as -log one-half R_max_; *t*-test: *P<0.05 vs. Untreated; ^#^P<0.05 *vs*. HgCl_2_-treated.

**Figure 3:**
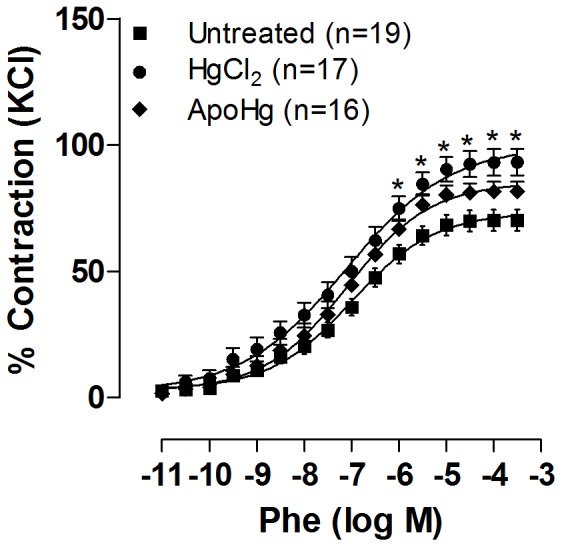
Effect of apocynin treatment on the vasoconstrictor response to phenylephrine. Concentration-response curve to phenylephrine (Phe) in the aortas of rats untreated, treated with mercury (HgCl_2_), and apocynin plus mercury (ApoHg). The results (mean±SEM) are expressed as a percentage of the response to 75 mmol/l KCl. The number of animals is indicated in parentheses. Two-Way ANOVA *P<0.05.

Vasoconstrictor responses to 75 mM KCl were similar (P>0.05) in aortas from mercury (1.52±0.24 g; n = 17), apocynin (1.48±0.27 g; n = 18), and apocynin-mercury (1.46±0.17 g; n = 16) groups compared to the untreated group (1.53±0.24 g; n = 19). In apocynin-treated rats, the response to phenylephrine was similar to that of untreated rats (Table 2). Treatment with mercury for 30 days increased the vasoconstrictor responses induced by phenylephrine in endothelium-intact aortic rings, as previously described [13], [17]. Co-treatment with apocynin partially prevented the increase in contractile response to phenylephrine in these arteries (Figure 3); thus, while mercury treatment increased the maximum response (R_max_), this increase was not observed in rats co-treated with apocynin (Table 2).

**Table 2:** Effects of apocynin treatment of rats in the absence and in the presence of HgCl_2_ on maximum response (R_max_) and sensitivity (pD2) to phenylephrine.

	Untreated		HgCl_2_		Apo		ApoHg	
	R_max_	pD2	R_max_	pD2	R_max_	pD2	R_max_	pD2
Control	75.6±5.4	−6.9±0.2	98.3±6.8^#^	−7.0±0.2	90.3±5.7	−8.5±1.4	84.7±4.9	−6.9±0.2
E-	135.5±5.9*	−14.8±1.9*	142.9±6.3*	−13.4±1.6*	139.9±4.9*	−14.2±2.5*	152.5±7.1*	−16.3±1.8*
L-NAME	159.3±6.7*	−10.3±1.4*	145.7±5.0*	−10.3±1.3*	155.7±8.1*	−11.8±1.6*	153.6±8.3*	−9.7±1.1*
Apocynin	31.8±4.6*	−6.6±0.1	25.8±4.7*	−6.5±0.2	37.0±5.7*	−6.6±0.2	40.9±6.4*	−6.7±0.2
Indomethacin	46.5±5.6*	−6.8±0.1	35.6±5.6*	−6.7±0.1	51.8±5.3*	−6.9±0.1	30.9±1.9*^#^	−6.7±0.2

Parameters of maximal response (Rmax) and sensitivity (pD2) of the concentration-response curves to phenylephrine in aortas from rats untreated, treated with mercury (HgCl_2_), apocynin and apocynin plus mercury (ApoHg) in intact (Control) and endothelium removal (E-) segments and in the presence of L-NAME (100 µM), Apocynin (0.3 mM) or Indomethacin (1 µM) incubation. Results are expressed as mean±SEM. R_max_, maximal effect (expressed as a percentage of maximal response induced by 75 mM KCl) and pD2 expressed as -log one-half R_max_; *t*-test: *P<0.05 compared to the corresponding control in each group; ^#^P<0.05 *vs*. Untreated group.

### Apocynin treatment diminishes the deleterious effect of mercury on endothelial NO modulation of vasoconstrictor responses

**Figure 4:**
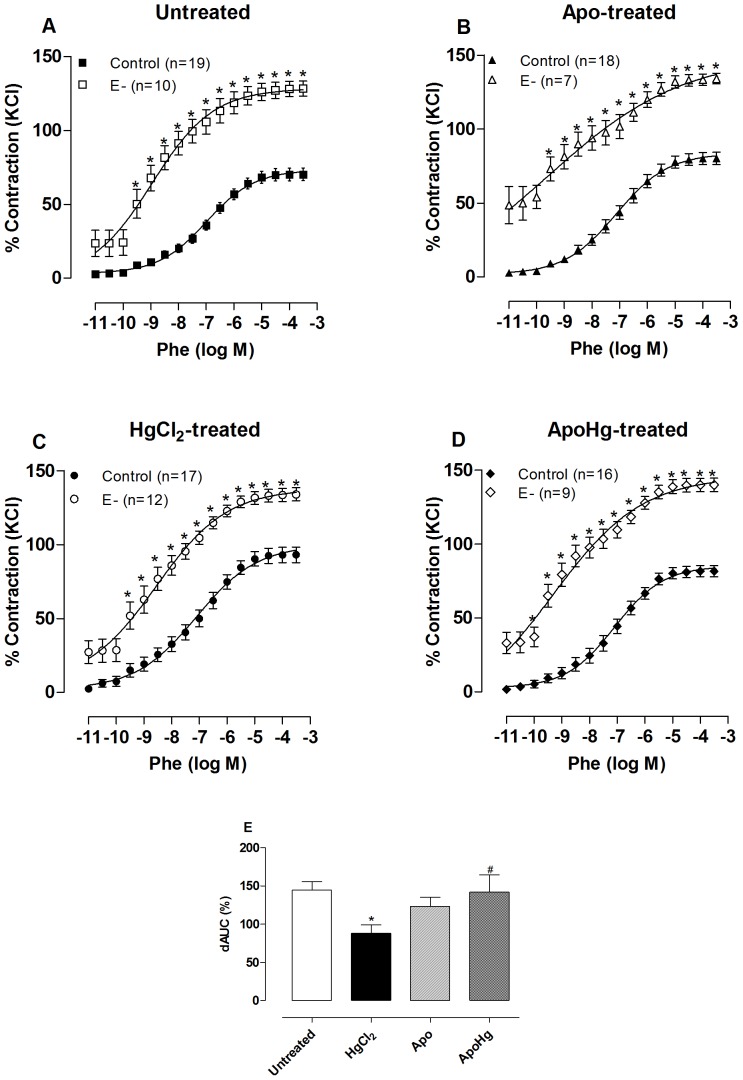
Effect of apocynin treatment on endothelial modulation of the vasoconstrictor response to phenylephrine. Concentration-response curve to phenylephrine (Phe) in intact (Control) and endothelium removal (E-) aortic segments of rats (A) untreated, (B) treated with apocynin (Apo), (C) mercury (HgCl_2_), and (D) apocynin plus mercury (ApoHg). The results (mean±SEM) are expressed as a percentage of the response to 75 mmol/l KCl. The number of animals is indicated in parentheses. *P<0.001 by Two-Way ANOVA. (E) Differences in the area under the concentration-response curves (dAUC) in endothelium denuded and intact segments of the four experimental groups. * P<0.05 *vs*. Untreated and ^#^ *vs*. HgCl_2_-treated by *t*-test.

Mechanical removal of the endothelium caused a significant increase in the phenylephrine response in all groups (Figure 4, Table 2). The effect of endothelium removal was smaller in mercury-treated animals, as shown by the dAUC values, indicating that mercury treatment reduced the negative modulation induced by the endothelium of the contractile response to phenylephrine. In apocynin-treated rats, the effect of endothelium removal was similar to untreated rats, while the co-treatment of mercury with apocynin (Figure 4E) prevented the reduction of endothelial modulation.

**Figure 5:**
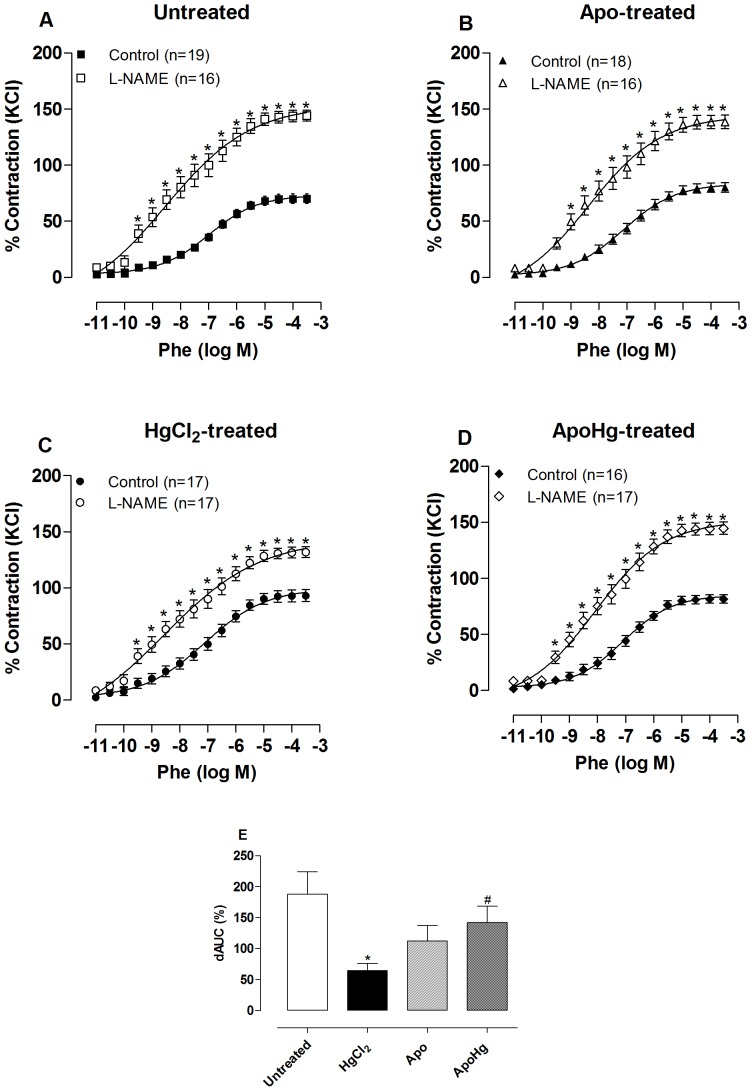
Effect of apocynin treatment on NO modulation of the vasoconstrictor response to phenylephrine. Concentration-response curve to phenylephrine (Phe) in aortic segments of rats (A) untreated, (B) treated with apocynin (Apo), (C) mercury (HgCl_2_), and (D) apocynin plus mercury (ApoHg) in the absence (Control) and the presence of the NO synthase inhibitor L-NAME (100 µM). The results (mean±SEM) are expressed as a percentage of the response to 75 mmol/l KCl. The number of rats is indicated in parentheses. *P<0.001 by Two-Way ANOVA. (E) Differences in the area under the concentration-response curve to phenylephrine (dAUC) in aortic segments in the presence and the absence of L-NAME of the four experimental groups. * P<0.05 *vs*. Untreated and ^#^ *vs*. HgCl_2_-treated by *t*-test.

To verify that the reduction of endothelial modulation in the vascular response to phenylephrine was due to alterations in the effects of NO, we used the NOS inhibitor L-NAME (100 µM) *in vitro*. This drug promoted an increase in the response to phenylephrine in arteries from both untreated and mercury-treated rats (Figure 5, Table 2). However, the dAUC values show that this increase was smaller in rats treated with mercury when compared to untreated (Figure 5E). Co-treatment with apocynin restored the potentiating effect of L-NAME in mercury-treated rats (Figure 5). In the group treated only with apocynin, L-NAME promoted increased sensitivity and maximal response to phenylephrine similar to that observed in the untreated group (Table 2). All of these results suggest that chronic treatment with low doses of mercury reduced NO bioavailability and its modulation of the contractile response to phenylephrine in rat aortas by the increase in superoxide anion production.

### Apocynin reduces the increased participation of ROS in vasoconstrictor responses of mercury-treated rats

**Figure 6:**
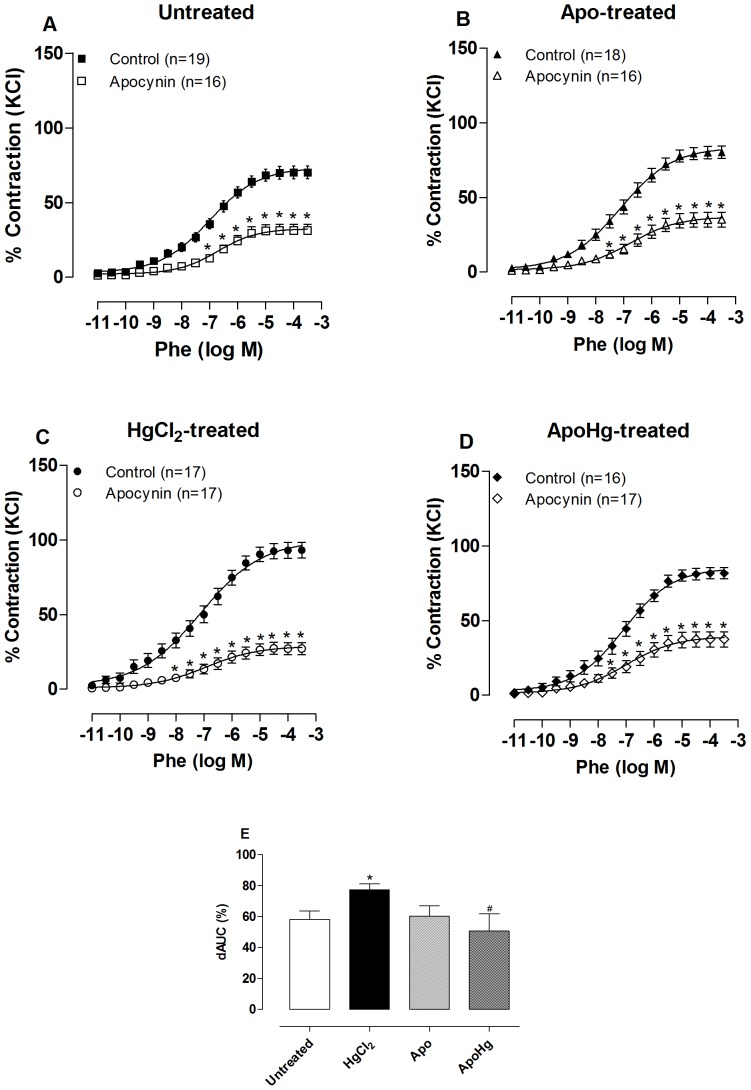
Effect of apocynin treatment on ROS modulation of the vasoconstrictor response to phenylephrine. Concentration-response curve to phenylephrine (Phe) in aortic segments of rats (A) untreated, (B) treated with apocynin (Apo), (C) mercury (HgCl_2_), and (D) apocynin plus mercury (ApoHg) in the absence (Control) and the presence of the NADPH oxidase inhibitor Apocynin (0.3 mM). The results (mean±SEM) are expressed as a percentage of the response to 75 mmol/l KCl. The number of rats is indicated in parentheses. *P<0.001 by Two-Way ANOVA. (E) Differences in the area under the concentration-response curve to phenylephrine (dAUC) in aortic segments incubated in the absence and the presence of apocynin of the four experimental groups. * P<0.05 *vs*. Untreated and ^#^ *vs*. HgCl_2_-treated by *t*-test.

To further evaluate the contribution of oxidative stress in the vascular alterations induced by mercury, we assessed the role of superoxide anion in phenylephrine responses using apocynin (0.3 mM) *in vitro*. This drug decreased the phenylephrine responses in arteries from both untreated and mercury-treated rats (Figure 6, Table 2). However, this inhibitory effect was higher in rats treated with mercury compared to the effect observed in untreated rats (Figure 6E). Co-treatment with apocynin restored the inhibitory effect of apocynin *(in vitro*) in mercury-treated rats. *In vitro* apocynin incubation in rats treated with apocynin induced a decrease in the response to phenylephrine similar to that observed in untreated rats.

### Effect of apocynin on lipid peroxidation and thiol groups in plasma

**Figure 7:**
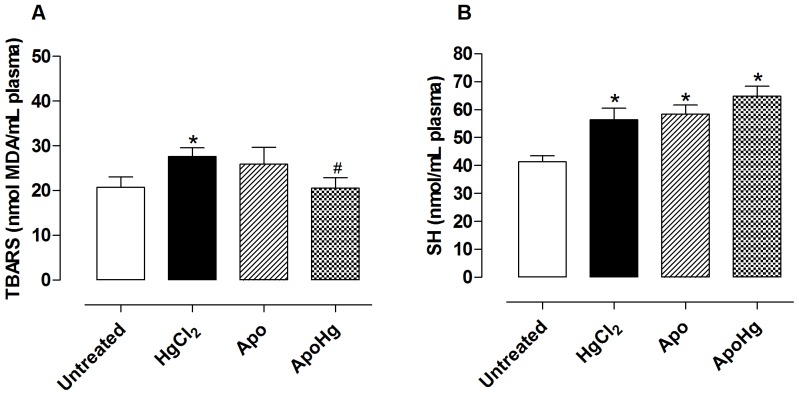
Effect of apocynin treatment on lipid peroxidation and thiol groups in plasma. Values of (A) TBARS and (B) thiol (SH) groups in the plasma of rats untreated (n = 7) and treated with mercury (HgCl_2_, n = 7), apocynin (Apo, n = 6) and apocynin plus mercury (ApoHg, n = 9). Data are expressed as the mean±SEM, *t*-test *P<0.05 *vs*. Untreated and ^#^P<0.05 *vs*. HgCl_2_-treated.

Chronic treatment with low doses of mercury promoted an increase in oxidative stress and lipid peroxidation, as demonstrated by increased plasma MDA levels. Apocynin co-treatment prevented the increase in plasma MDA levels (Figure 7A), suggesting that the superoxide anion produced by exposure to mercury might be the main ROS involved in this oxidative stress.

Mercury treatment also increased the plasma levels in SH groups (Figure 7B). Co-treatment with apocynin did not alter the increase of thiol groups resulting from exposure to the metal. In addition, SH groups were increased in the apocynin group (Figure 7B).

### Effect of apocynin on superoxide dismutase (SOD) and glutathione peroxidase (GPx) activity

**Figure 8:**
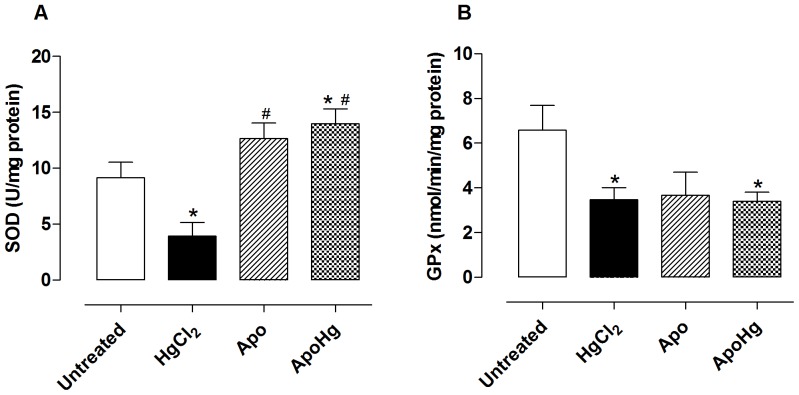
Effect of apocynin treatment on SOD and GPx activity. Values of (A) SOD and (B) GPx activities in the aortas of rats untreated (n = 6) and treated with mercury (HgCl_2_, n = 6), apocynin (Apo, n = 6) and apocynin plus mercury (ApoHg, n = 6). Data are expressed as the mean±SEM. *t*-test *P<0.05 *vs*. Untreated and ^#^P<0.05 *vs*. HgCl_2_-treated.

Chronic exposure to low doses of mercury caused a reduction in aortic SOD activity (Figure 8A). Apocynin not only prevented this reduction, but it also increased the enzyme activity when compared to untreated animals both in the absence and presence of mercury (Figure 8A). In rats exposed to mercury for 30 days, a reduction in aortic GPx activity was also observed (Figure 8B). Apocynin failed to prevent this reduction caused by the metal (Figure 8B). This result suggests that apocynin does not act on the GPx pathway.

### Apocynin treatment does not modify the participation of prostanoids on vasoconstrictor responses in mercury-treated rats

**Figure 9:**
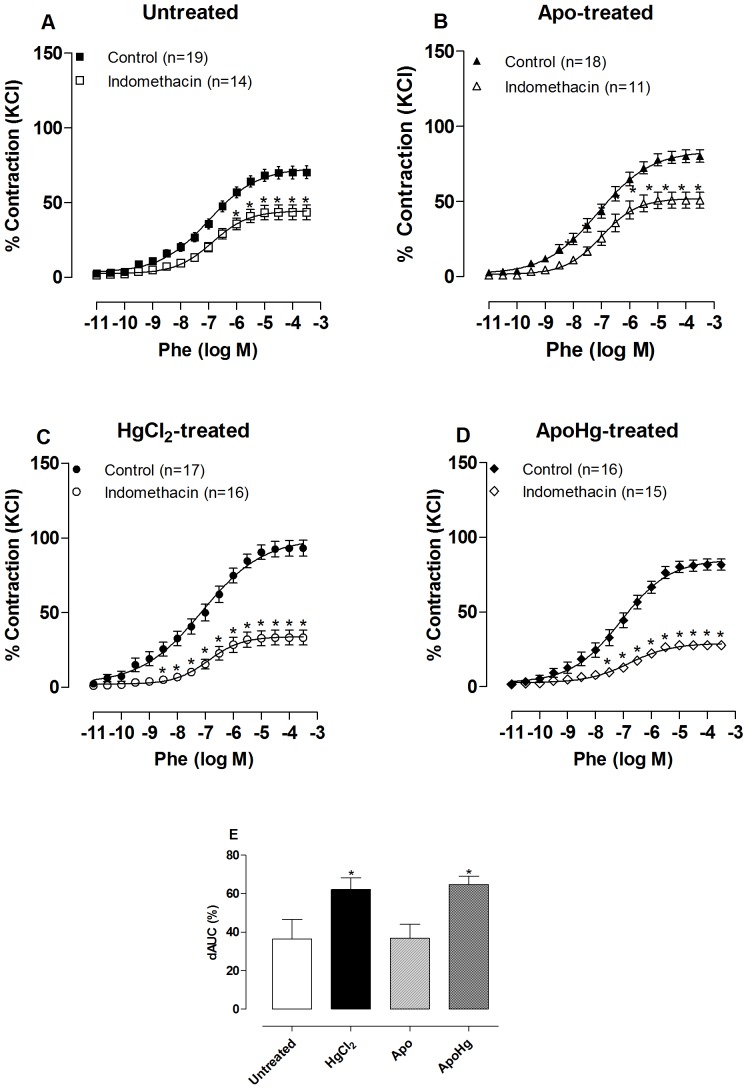
Effect of apocynin treatment on prostanoid modulation of the vasoconstrictor response to phenylephrine. Concentration-response curve to phenylephrine (Phe) in aortic segments of rats (A) untreated, (B) treated with apocynin (Apo), (C) mercury (HgCl_2_), and (D) apocynin plus mercury (ApoHg) in the absence (Control) and the presence of the non-selective COX inhibitor Indomethacin (1 µM). The results (mean±SEM) are expressed as a percentage of the response to 75 mmol/l KCl. The number of rats is indicated in parentheses. *P<0.001 by Two-Way ANOVA. (E) Differences in the area under the concentration-response curve to phenylephrine (dAUC) in aortic segments in the absence and the presence of indomethacin of the four experimental groups. * P<0.05 *vs*. Untreated by *t*-test.

Previous studies have shown that the increased participation of COX vasoconstrictor prostanoids may be associated with oxidative stress, hypertension and increased vascular reactivity [22], [23]. We found that the COX inhibitor indomethacin (1 µM) reduced the response to phenylephrine more in arterial segments of mercury-treated than untreated rats (Figure 9, Table 2), as previously described [13]. Apocynin co-treatment of mercury-treated rats did not modify the effect of indomethacin on phenylephrine responses. These results suggest that in rats treated with mercury, there is greater participation of vasoconstrictor prostanoids derived from COX on the vasoconstrictor response to phenylephrine, which was not dependent on the observed increased oxidative stress.

## Discussion

The present study shows, for the first time, that apocynin treatment normalizes endothelial dysfunction in the aortas of rats chronically exposed to low doses of mercury chloride. This effect is likely due to its action on NADPH oxidase, preventing mercury-induced oxidative stress, as demonstrated by decreased plasma MDA and the increased activity of SOD, resulting in increased NO bioavailability in the aortic tissue. Our results also show that mercury increases phenylephrine responses by acting on oxidative stress and COX pathways. A blockade of NADPH oxidase partially prevents the mercury-induced phenylephrine hyperresponsiveness by reducing oxidative stress without affecting COX pathways.

Mercury has been identified as a hazard and risk factor for cardiovascular disease in humans [10]. The United States Environmental Protection Agency recommends a maximum blood concentration of mercury of 5.8 ng/ml; below this concentration, mercury exposure is considered to be without adverse effects [3], [24]. Several studies have shown blood mercury level in residents of contaminated areas and in mercury-exposed workers ranging from 7 to 10 ng/ml [25]. In the present study, we used a controlled low-dose administration of mercury chloride and attained a blood mercury content of approximately 8 ng/ml [17], close to the human exposure levels.

It has been shown that mercury exposure increases the risk of hypertension, carotid atherosclerosis and coronary heart disease [26]. We have recently described that 30 days of exposure to low doses of HgCl_2_ produces important vascular dysfunction in the aorta, coronary and mesenteric resistance arteries [12]–[14]. These effects can be explained, at least in part, by the oxidative stress caused by mercury, which is responsible for increasing the production of ROS, reduction of NO bioavailability and endothelial dysfunction. In fact, oxidative stress is well documented in mercury poisoning as demonstrated by increased lipid peroxidation [27] and decreased antioxidant defenses of the body [28], [29]. The damage caused by this metal on the cardiovascular system can be attributed to the activation of many ROS sources, but to our knowledge, this is the first study demonstrating the essential role of NADPH oxidase in mercury-induced vascular dysfunction.

The effects caused by mercury exposure on blood pressure vary with the dose of metal and exposure time. Acute administration of a high concentration of mercury decreases arterial blood pressure in rats, [30], [31] while chronic treatment showed the opposite effect [9], [32]. However, exposure to nanomolar doses of mercury does not promote changes in blood pressure [11]–[14]. The results obtained in this study confirm and extend previous findings [17], demonstrating that there are no changes in systolic and diastolic blood pressure by exposure to low doses of mercury. Interestingly, apocynin reduced systolic and diastolic blood pressures in the control rats, but it did not do so in the mercury-treated rats. We do not have a clear explanation of these findings, but possibilities include higher resistance to apocynin effects in mercury-treated animals by affecting other organs involved in blood pressure control such as the kidneys or heart. Future studies on these issues will clarify this point.

The exposure to mercury impaired vasodilator responses to acetylcholine, as previously shown [11]–[13]. Because the vascular response to sodium nitroprusside was unaffected by mercury, these findings suggest the presence of endothelial dysfunction secondary to the metal exposure, independent of smooth muscle sensitivity to NO. Apocynin administration normalized the impaired acetylcholine response in mercury-treated rats, suggesting that vascular NADPH oxidase plays a role in the endothelial dysfunction of conductance arteries in rats chronically exposed to low concentrations of mercury. The ability of apocynin to improve endothelial-dependent vasodilator responses has been described in different hypertension models [23], [33], [34]. In addition, we have previously demonstrated that when administered *in vitro* in the organ bath, apocynin also improved the impaired response to acetylcholine in aortas from mercury-treated rats [12].

In our study, we also observed an increase in vascular reactivity to phenylephrine in aortas of rats exposed to mercury. Similar findings were reported using either high or low mercury concentrations *in vitro* [11], [17]. Furthermore, we observed that endothelium removal abolished this effect, suggesting that mercury produces changes at the endothelial level. This finding is reinforced by the mentioned decrease of the endothelium-dependent vasodilator response induced by acetylcholine. Importantly, co-treatment with apocynin prevents the above-mentioned changes, suggesting that mercury acts mainly through the activation of NADPH oxidase by affecting the endothelial modulation of phenylephrine responses. More specifically, mercury decreased NO availability, as demonstrated by the effect of L-NAME on phenylephrine responses being smaller in mercury-treated animals than in untreated animals and apocynin normalizing this effect.

As expected, the apocynin treatment of rats prevented the increased participation of superoxide anion in the contractile response to phenylephrine in the aortas of rats chronically exposed to HgCl_2._ Previous studies have demonstrated that apocynin reverses endothelial NO dysfunction in animals or humans with elevated levels of oxidative stress [35]. Other authors have reported a protective effect of *in vivo* treatment with apocynin in experimental models of vascular injury associated with ROS overproduction [36]–[38]. Therefore, apocynin appears to be an effective NADPH oxidase inhibitor *in vivo* and acts to prevent vascular damage associated with oxidative stress increases such as chronic mercury exposure.

Although apocynin co-treatment affected phenylephrine responses, only a partial prevention of the mercury-induced effect was observed, indicating that other endothelial factors are also involved in the generation of the harmful effects induced by HgCl_2_ intoxication. Indomethacin, a non-selective COX inhibitor, reduced the contractile response to phenylephrine in segments from all groups, but this reduction was greater in the group treated with mercury, showing that in this group, there was increased participation of the COX pathway in vascular responses. In agreement, we previously demonstrated the involvement of COX-derived vasoconstrictor prostanoids in the vascular effects of high concentrations of HgCl_2_ [11] or in the same model as used here [13]. Some studies have demonstrated the existence of a reciprocal feed-forward relationship between the NADPH oxidase COX pathway in hypertension [22], [34], [39]. However, in our experimental conditions, co-treatment with apocynin did not modify the increased participation of the COX pathway in the contractile response to phenylephrine caused by mercury, indicating that activation of COX seems independent of oxidative stress generated by NADPH oxidase.

Exposure to HgCl_2_ increases the levels of thiol (SH) groups as well as plasma lipid peroxidation, as evidenced by the increased levels of MDA. It has been described that Hg^2+^ reacts with SH groups, thus depleting intracellular thiols, especially glutathione (GSH), and causing cellular oxidative stress or predisposing cells to it [40] and forming free radicals that may further increase lipid peroxidation. However, in our experimental conditions, the increase in SH groups caused by mercury may be related to the time and concentration used and may generate a compensatory mechanism.

The increase in oxidative stress may be due to a decrease in the antioxidant defenses or to an increase in the production of ROS [41], [42]. Our study showed that exposure to mercury also reduced SOD and GPx activities. Similarly, Benov *et al*. [43] showed that mercury inhibits the activity of several antioxidant enzymes such as CAT, SOD and GPx in the blood, liver and kidneys of mercury-poisoned rats. It was also demonstrated that GPx and SOD activities were significantly lower in erythrocytes from occupationally-exposed workers [44]. Recently, it was further demonstrated that chronic exposure to high doses of methylmercury in drinking water reduced the activity of selenoproteins such as GPx in the cortex and cerebellum of mice, which was attributed to reduced gene expression of these enzymes [45]. In addition, Zalups *et al*. [7] observed that exposure to mercury may also reduce non-enzymatic antioxidant defenses, such as GSH, the major low-molecular-mass thiol compound present in virtually all mammalian tissues [46]. In contrast, in another study with occupational exposure to mercury in elementary form (Hg), increases in GSH, glutathione reductase (GR) and CAT activity were observed in erythrocytes from miners [8], which have been attributed to compensatory mechanisms due to low levels of exposure. The co-treatment with apocynin not only prevented the reduction in SOD activity caused by mercury in aortas but also promoted an increase in its activity. However, this was not the case for GPx, suggesting that NADPH oxidase-derived ROS are able to modulate SOD activity at the vascular level.

In summary, our results demonstrate for the first time that the NADPH oxidase inhibitor apocynin can partially prevent the increase in vascular reactivity to phenylephrine and improve the endothelial dysfunction in aortas of rats chronically exposed to nanomolar concentrations of mercury. This effect is due to the prevention of oxidative stress, lipid peroxidation and depletion of defense mechanisms caused by this metal. In addition, we demonstrated that mercury acts using two different and independent pathways, i.e., NADPH oxidase and COX, whereas only the NADPH oxidase pathway is affected by apocynin treatment. These results suggest that the therapeutic use of apocynin in preventing deleterious effects on the cardiovascular system caused by occupational exposure to mercury may have important clinical implications and should continue to be extensively studied.
